# Bridging inflammation and proliferation: scRNA-seq analysis of chemotactic and growth factor signaling in mouse skin wound repair

**DOI:** 10.3389/fimmu.2025.1654043

**Published:** 2025-09-25

**Authors:** Lingzhang Meng, Hongmian Li, Jian Song, Wenxian Lin, Xiuli Mao, Xiamin Zhang, Mingyue Yang, Kezhao Wu, Liu Lu, Feiteng Liang, Feng Long, Yueyong Li, Qiang Tang

**Affiliations:** ^1^ Institute of Cardiovascular Sciences, Guangxi Academy of Medical Sciences & The People’s Hospital of Guangxi Zhuang Autonomous Region, Nanning, China; ^2^ Department of Emergency, Guangxi Academy of Medical Sciences & The People’s Hospital of Guangxi Zhuang Autonomous Region, Nanning, China; ^3^ Graduate School, Youjiang Medical University for Nationalities, Baise, China; ^4^ Department of Pharmacology, Youjiang Medical University for Nationalities, Baise, China; ^5^ Department of Engineering, The University of Sydney, Sydney, NSW, Australia; ^6^ Key Laboratory of Medical Research Basic Guarantee for Immune-related Diseases Research of Guangxi (Cultivation), Affiliated Hospital of Youjiang Medical University for Nationalities, Baise, China; ^7^ Burn Plastic & Trauma Surgery Department, Affiliated Hospital of Youjiang Medical University for Nationalities, Baise, China; ^8^ Department of Infectious Diseases, The People’s Hospital of Beihai, Beihai, China; ^9^ Department of Oncology, Changsha Central Hospital, University of South China, Changsha, China

**Keywords:** wound healing, scRNA-seq, chemotactic signaling, VEGF and EGF pathways, mouse skin model

## Abstract

**Introduction:**

The transition from inflammation to proliferation is a critical but poorly understood phase in wound healing. To elucidate the cellular and molecular dynamics of this pivotal stage, we performed single-cell RNA sequencing (scRNA-seq) on mouse skin biopsies 4 days after injury.

**Methods:**

By employing our newly developed R packages, OptiRes for optimized clustering and TidyGenePlot for annotation, we identified 21 distinct cell types. CellChat analysis was used to identify intercellular communication clusters. Findings on chemotactic signaling through CCR5, CCR1, and ACKR1 were validated in vivo, and the functional significance was confirmed by demonstrating that inhibition of CCR pathways reduced phagocyte infiltration.

**Results:**

Our analysis revealed a dynamic shift in cellular composition, characterized by an influx of neutrophils, classical monocytes, and M1 macrophages. This recruitment of phagocytes was driven by enhanced chemotactic signaling through CCR5, CCR1, and ACKR1. Furthermore, CellChat analysis identified four distinct intercellular communication clusters, highlighting the early activation of VEGF and EGF signaling pathways, which are essential for angiogenesis and re-epithelialization.

**Discussion:**

Together, these findings provide a high-resolution map of the cellular and molecular landscape during the transition from inflammation to proliferation, offering novel insights into the mechanisms that orchestrate tissue repair and identifying potential intervention manner to enhance wound healing.

## Introduction

Wound healing is a fundamental physiological process involving a coordinated sequence of inflammation, proliferation, and remodeling (1). While essential for tissue repair, the transition from the late inflammatory phase to the proliferative phase remains a critical yet poorly understood window that largely determines healing outcomes (2). Single-cell RNA sequencing (scRNA-seq) offers an unprecedented opportunity to dissect the cellular heterogeneity and molecular signaling within the wound microenvironment, enabling a high-resolution analysis of this pivotal transition.

Previous research has established the roles of key cellular players: immune cells, such as neutrophils and macrophages (3, 4), orchestrate the initial inflammatory response, while growth factors like VEGF and EGF drive subsequent angiogenesis and re-epithelialization (3, 5). However, the precise molecular mechanisms that bridge these two phases—specifically, how proliferative pathways are primed while inflammation is still resolving—remain to be elucidated (6). The mouse skin wound model, particularly at 4 days post-injury, provides an ideal system for investigating this overlap, as it captures the dynamic interplay between resolving inflammation and initiating proliferation (7, 8).

In this study, we leveraged the scRNA-seq count matrix of mouse skin at this critical 4-day time point to map the cellular and molecular landscape of the inflammation-to-proliferation transition. To overcome challenges in data analysis and visualization, we developed two novel R packages: OptiRes, for determining the optimal clustering resolution, and TidyGenePlot, to help annotate cell types. Using this robust analytical pipeline, we aimed to comprehensively characterize the cellular composition, map the intercellular communication networks, and identify the key signaling pathways that orchestrate this transitional phase. Ultimately, this work seeks to provide a detailed blueprint of the mechanisms governing the switch from inflammation to proliferation, uncovering potential intervention manner to improve tissue repair.

## Materials and methods

### Animal model and sample collection

All experiments were conducted using 8–12-week-old C57BL/6J mice housed under specific pathogen-free conditions with a 12-hour light/dark cycle and ad libitum access to food and water. Full-thickness excisional wounds (6 mm diameter) were created on the dorsal skin under isoflurane anesthesia (9), and biopsies were collected at 4 days post-wounding, corresponding to the late inflammation to early proliferation transition, along with unwounded control skin samples (7, 8). Tissue samples were immediately snap-frozen in liquid nitrogen and stored at -80°C until processing. All the animal experiments were conducted under the IACUC guidelines and were approved by the Ethics Committee of Youjiang Medical University for Nationalities (Approval NO. 2021030301).

### Intervention with antagonist cocktail

The intervention involved the administration of a cocktail comprising BX471 (a CCR1 antagonist) (10), Maraviroc (a CCR5 antagonist) (11), and Blocking Peptide LS-E41165 (an ACKR1 inhibitor, purchased from LS Bio) to wounded mice. The cocktail was prepared by dissolving BX471 (10 mg/kg), Maraviroc (5 mg/kg), and Blocking Peptide LS-E41165 (2 mg/kg) in a vehicle solution of 10% dimethyl sulfoxide (DMSO) and 90% saline, ensuring a final volume of 200 µL per dose. The solution was administered intraperitoneally daily for 3 consecutive days, starting 24 hours post-wounding, using a 1 mL syringe with a 26-gauge needle. Control mice received an equivalent volume of saline solution. Efficacy was evaluated by collecting skin biopsies 4 days post-wounding, followed by flow cytometry analysis to assess CD45+CD11b+ phagocyte populations in treated versus untreated groups.

### Tissue preparation for histological analysis

Peri-wound skin samples were collected from mice at specified time points (e.g., Day 12 post-injury) and fixed in 4% paraformaldehyde (PFA) in phosphate-buffered saline (PBS) for 24 hours at 4°C. Fixed tissues were then dehydrated through a graded ethanol series (70%, 95%, 100%), cleared in xylene, and embedded in paraffin wax. Serial sections (5 μm thick) were cut using a microtome (Leica RM2255) and mounted on glass slides for subsequent staining.

### Hematoxylin and eosin staining

Paraffin-embedded sections were deparaffinized in xylene (two changes, 5 minutes each) and rehydrated through a descending ethanol series (100%, 95%, 70%, 50%, distilled water; 2 minutes each). Sections were stained with Harris hematoxylin (Sigma-Aldrich) for 5 minutes to label nuclei, followed by rinsing in tap water for 5 minutes to develop the blue color. Differentiation was performed in 1% acid alcohol (1% HCl in 70% ethanol) for 5–10 seconds, and sections were then blued in Scott’s tap water substitute (0.35% sodium bicarbonate, 2% magnesium sulfate in water) for 1 minute. Counterstaining was achieved with eosin Y (1% in 95% ethanol; Sigma-Aldrich) for 2 minutes to highlight cytoplasmic and extracellular components. Sections were dehydrated through an ascending ethanol series (70%, 95%, 100%; 2 minutes each), cleared in xylene (two changes, 2 minutes each), and mounted with a coverslip using DPX mounting medium (Sigma-Aldrich). Stained sections were imaged using a bright-field microscope (Olympus BX53) at 10× and 20× magnification. Re-epithelialization was quantified by measuring the epithelial gap distance using ImageJ software (NIH), with data expressed as mean ± standard deviation from at least three independent fields per sample.

### Masson’s trichrome staining

Deparaffinized and rehydrated sections (as described above) were mordanted in Bouin’s solution (Sigma-Aldrich) for 1 hour at 56°C to enhance staining affinity. Sections were rinsed in running tap water until clear and stained with Weigert’s iron hematoxylin (Sigma-Aldrich) for 10 minutes to label nuclei black. After rinsing in distilled water, sections were differentiated in 1% acid alcohol for 5 seconds and rinsed again. Staining proceeded with Biebrich scarlet-acid fuchsin solution (Sigma-Aldrich) for 10–15 minutes to label cytoplasm and muscle fibers red, followed by rinsing in distilled water. Differentiation and collagen staining were achieved by immersion in phosphomolybdic-phosphotungstic acid (Sigma-Aldrich) for 10–15 minutes, then aniline blue (Sigma-Aldrich) for 5 minutes to label collagen blue. Sections were differentiated in 1% acetic acid for 2–5 minutes, dehydrated through an ascending ethanol series (95%, 100%; 2 minutes each), cleared in xylene (two changes, 2 minutes each), and mounted with DPX. Imaging was performed using a bright-field microscope (Olympus BX53) at 10× and 20× magnification. ECM remodeling was quantified by measuring the percentage of blue-stained collagen area relative to total tissue area using ImageJ software, with results averaged from multiple fields per section.

### Immunofluorescence imaging

Deparaffinized and rehydrated sections were subjected to antigen retrieval by heating in citrate buffer (10 mM sodium citrate, pH 6.0) at 95°C for 20 minutes, followed by cooling to room temperature. Non-specific binding was blocked with 5% bovine serum albumin (BSA; Sigma-Aldrich) in PBS containing 0.1% Tween-20 (PBST) for 1 hour at room temperature. Sections were incubated overnight at 4°C with primary antibodies: rabbit anti-Ki67 (1:200 dilution; Abcam, ab15580) for proliferation, and goat anti-CD31 (1:100 dilution; R&D Systems, AF3628) for endothelial cells. After washing three times with PBST (5 minutes each), sections were incubated with secondary antibodies: Alexa Fluor 594-conjugated donkey anti-rabbit (1:500; Thermo Fisher, A-21207) for Ki67 (red) and Alexa Fluor 488-conjugated donkey anti-goat (1:500; Thermo Fisher, A-11055) for CD31 (green) for 1 hour at room temperature in the dark. Nuclei were counterstained with 4’,6-diamidino-2-phenylindole (DAPI; 1 μg/mL; Sigma-Aldrich) for 5 minutes. Sections were washed three times with PBST, mounted with ProLong Gold antifade reagent (Thermo Fisher), and imaged using a fluorescence microscope (Zeiss Axio Observer) with appropriate filters for DAPI (blue), Alexa Fluor 488 (green), and Alexa Fluor 594 (red) at 20× and 40× magnification. Quantitative analysis was performed using ImageJ software: Ki67+/CD31+ co-localized cells were counted per mm², and relative fluorescence intensity (e.g., CD31/DAPI) was measured from at least five random fields per sample. Data were analyzed for statistical significance using unpaired t-tests in GraphPad Prism (version 9.0).

### Bioinformatic analysis

Single-cell RNA sequencing (scRNA-seq) data were downloaded from NCBI Gene Expression Omnibus (GEO) under accession number GSE142471 (12). Bioinformatic processing was performed using the Seurat R package (version 5.3.0) (13), where count matrices were subjected to quality control by excluding cells with fewer than 200 or more than 2500 unique molecular identifiers (UMIs) or with mitochondrial gene content exceeding 10%. Data were normalized using the SCTransform method, and the top 3000 highly variable genes were selected for downstream analysis. Principal component analysis (PCA) was conducted, and the first 20 principal components were used for uniform manifold approximation and projection (UMAP) dimensionality reduction. Cell type annotation was performed using feature plots generated with the TidyGenePlot R package (version 1.0.0) developed by our team, overlaid on the UMAP projection, with canonical gene markers used to identify cell types. Hierarchical clustering was conducted using Ward’s method to generate dendrograms visualizing transcriptional relationships. Cell-cell interaction networks were analyzed using the CellChat R package (version 2.2.0) (14), with interaction strengths inferred from ligand-receptor pair expression profiles across the 21 cell types, compared between control and wounded samples using a scatter plot.

### Determination of the optimal clustering resolution

Optimal clustering resolution was determined using the R package OptiRes (version 1.0.0) developed by our team, which calculates the silhouette score (15, 16) for each resolution using the formula:


$$S(i)=\frac{b(i)-a(i)}{\text{max}(a(i),b(i))}$$

The final Silhouette Score for the entire clustering is the average of all S(i) values for all data points:


$$S=\frac{1}{n}\sum_{i=1}^{n}\;S(i)$$

### Chemotactic signaling analysis

Chemotactic signaling was assessed by flow cytometry and computational analysis of ligand-receptor interactions. For flow cytometry, single-cell suspensions were prepared from control and wounded skin biopsies as described above. After blocking with Fcγ blocker, cells were stained with the following fluorochrome-conjugated antibodies: anti-CD45 (clone 30-F11), anti-CD11b (clone M1/70anti-Ly6G (clone 1A8), anti-Ly6C (clone HK1.4), and anti-F4/80 (clone BM8), all diluted 1:200 in PBS with 2% FBS. Staining was performed at 4°C for 30 minutes in the dark, followed by washing and resuspension in PBS. Samples were analyzed using a BD LSRFortessa flow cytometer, with at least 50,000 events collected per sample. Data were processed using FlowJo software (version 10.8.1) to quantify CD45+CD11b+ phagocyte populations, including neutrophils (Ly6G+), classical monocytes (Ly6C+), and M1 macrophages (F4/80+), with relative frequencies compared between conditions. Bar graphs and circular plots, generated with the ggplot2 R package, depicted the interaction strengths of ligand-receptor pairs (*CCL3*, *CCL5*, *CCL7*, *CXCL1*, *CXCL2*) mediated by CCR5, CCR1, and ACKR1, inferred from scRNA-seq data using CellChat. Interventions involved intraperitoneal administration of a cocktail (BX471, Maraviroc, and Blocking Peptide LS-E41165) daily for 3 days, with efficacy evaluated by flow cytometry of treated versus untreated wounded skin samples.

### Statistical analysis

Statistical comparisons of cell frequencies and interaction strengths were performed using unpaired two-tailed Student’s t-tests, with significance levels denoted as ns (not significant), *p < 0.05, **p < 0.01, and ***p < 0.001. All analyses were based on three independent experiments, with individual readouts represented as dots in statistical dot plots. Data are presented as mean ± standard deviation, and all statistical tests were conducted using Graphpad Prism.

## Results

### Temporal dynamics of immune cell infiltration and endothelial proliferation in murine wound healing

The temporal dynamics of neutrophil and classical monocyte infiltration, as well as endothelial cell proliferation, were investigated in peri-wound skin during murine wound healing. Representative flow cytometry plots (**Supplementary Figure 1A**) demonstrated a decreasing tendency of neutrophils and classical monocytes within the CD45+CD11b+ populations in peri-wound skin samples at Day 2, Day 4, and Day 6 post-injury. Quantitative analysis (**Supplementary Figure 1B**) revealed that classical monocyte (Ly6G-Ly6C+) frequencies decreased significantly over time, with values of approximately 12.5% at Day 2, 9.7% at Day 4, and 5.2% at Day 6. Similarly, neutrophil (Ly6G+Ly6C-) frequencies declined from 50.3% at Day 2 to 29.6% at Day 4 and 6.4% at Day 6. The inflammatory phase usually lasts from day 1 to about day 3 or 4 after the injury (17), involving neutrophils followed by macrophages, which clear bacteria and debris while releasing growth factors.

Immunofluorescence staining of Ki67 (red), CD31 (green), and DAPI (blue) in peri-wound skin samples (**Supplementary Figure 1C**) illustrated endothelial cell proliferation, marked by Ki67 and CD31 co-expression, at Day 2, Day 4, and Day 6 post-injury. Quantitative analysis of Ki67+/CD31+ cell numbers per mm² (**Supplementary Figure 1D**) indicated a significant increase over time, with values of approximately 200 cells/mm² at Day 2, 600 cells/mm² at Day 4, and 800 cells/mm² at Day 6. By day 4, the proliferation phase are becoming dominant. This phase focuses on covering the wound aniogenesis, forming new tissue (granulation tissue). These findings suggest that Day 4 post-wound represents a transitional phase from inflammation to proliferation, as evidenced by the decline in inflammatory cell frequencies and the concurrent rise in endothelial cell proliferation.

This analysis provides a comprehensive understanding of the dynamic cellular changes during murine wound healing, highlighting the pivotal role of Day 4 as a transition point between these two critical phases.

### Comprehensive analysis of cell type identification and cell-cell interactions in mouse skin

**Figure 1:**
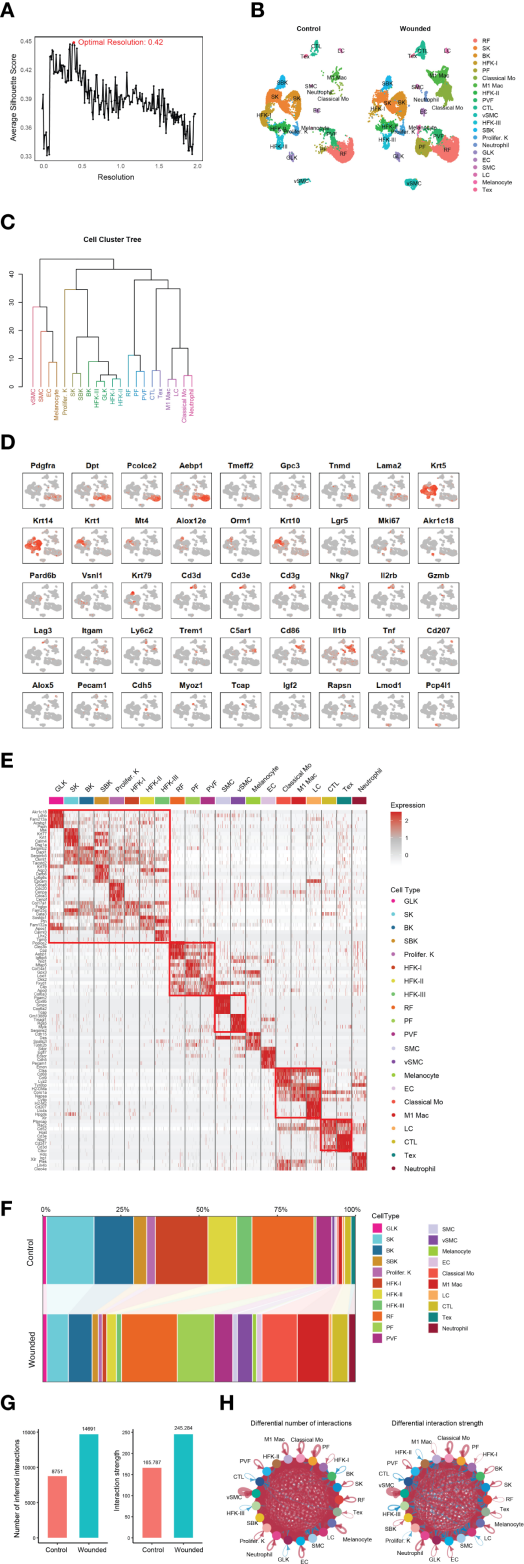
Comprehensive analysis of cell type identification and dynamics in control and wounded mouse skin. **(A)** Silhouette score analysis used to determine the optimal clustering resolution (e.g., 0.42 shown) for downstream cell type identification. **(B)** UMAP plot illustrating the distribution of identified cell types from control and wounded mouse skin samples. **(C)** Dendrogram depicting the hierarchical relationships among the 21 annotated cell types (annotated). **(D)** Feature plots showing the expression of canonical gene markers for various cell types overlaid on the UMAP projection. Markers include: Pan-Fibroblast (*Pdgfra*, *Dpt*); Reticular Fibroblast (*Pcolce2*, *Aebp1*); Papillary Fibroblast (*Tmeff2*, *Gpc3*); Perivascular Fibroblast (*Tnmd*, *Lama2*); Pan-Keratinocytes (*Krt5*, *Krt14*); Spinous Keratinocytes (*Krt1*, *Mt4*); Suprabasal Keratinocytes (*Alox12e*, *Orm1*, *Krt10*); HFH-I (*Lgr5*); Proliferating Keratinocytes (*Mki67*); Granular Layer Keratinocytes (*Akr1c18*); Basal Keratinocytes (*Pard6b*); HFK-II (*Vsnl1*), HFK-II (*Krt79*); General CTL (*Cd3d*, *Cd3e*, *Cd3g*, *Nkg7*); Activating T cells (*Il2rb*); Effector T cells (*Gzmb*); Exhausted T cells (Tex; *Lag3*); Classical Monocytes (Mo; *Itgam*, *Ly6c2*); Neutrophils (*Trem1*, *C5ar1*); M1 Macrophages (*Cd86*, *Il1b*, *Tnf*); Langerhans Cells (LC; *Cd207*, *Alox5*); Endothelial Cells (EC; *Pecam1*, *Cdh5*); Smooth Muscle Cells (SMC; *Myoz1*, *Tcap*); Melanocytes (*Rapsn*); and Vascular Smooth Muscle Cells (VSMC; *Lmod1*, *Pcp4l1*). **(E)** Heatmap displaying Differentially Expressed Genes (DEGs) between wounded and control conditions across identified cell clusters. **(F)** Stacked bar plot illustrating changes in cellular composition (proportions of cell types) between wounded and control mouse skin biopsies. **(G)** Bar plots summarizing overall cell-cell interaction numbers and strengths in wounded versus control skin. **(H)** Circular plots depicting differential cell-cell interaction numbers and strengths between specific cell types in wounded versus control skin.

To investigate the cellular heterogeneity and molecular dynamics of epidermal wound healing, we analyzed single-cell RNA sequencing (scRNA-seq) data from mouse skin biopsies (NCBI GEO: GSE142471), comparing control (unwounded) and wounded conditions four days post-injury. Using Seurat clustering, we identified 21 distinct cell types at an optimal resolution of 0.42 (**Figure 1A**), as determined by silhouette score analysis with the OptiRes R package developed by our team. The hierarchical organization of these unannotated clusters is shown in **Supplementary Figure 2A**, with their distribution visualized in a UMAP plot (**Supplementary Figure 2B**). A UMAP projection visualizes the distribution of 21 annotated cell types in both control and wounded mouse skin samples, highlighting their spatial organization (**Figure 1B**). The hierarchical relationships among these cell types, including reticular fibroblasts (RF), papillary fibroblasts (PF), basal keratinocytes (BK), and immune cells such as classical monocytes (Mo) and M1 macrophages, are depicted in a dendrogram (**Figure 1C**). The dendrogram, derived from gene expression profiles, exhibits a multi-level branching pattern with dissimilarity heights ranging from 0 to 40, reflecting transcriptional distances between cell types (18). Major clusters form at higher heights (e.g., 30–40), with finer subdivisions at lower heights (e.g., 10–20), consistent with hierarchical clustering methods such as Ward’s or average linkage. Vascular smooth muscle cells (vSMC) and neutrophils, with longer branches, show greater dissimilarity, aligning with their distinct roles in vascular support and immune response. Conversely, hair follicle keratinocytes (HFK-I, HFK-II, HFK-III) form a tighter cluster with shorter branches (height ~10–15), supporting their shared keratinocyte lineage. The grouping of related cell types, such as keratinocytes (BK, spinous keratinocytes [SK]) and immune cells (Mo, M1 macrophages), is biologically plausible, reflecting their embryological origins and functions. This structure is scientifically sound, as it aligns with established cellular hierarchies in skin biology and is supported by the use of canonical gene markers (**Figure 1D**). However, the precise ordering of rare cell types (e.g., melanocytes) warrants caution due to potential sampling variability, and statistical validation of branch stability could further enhance confidence.

Feature plots, generated using the TidyGenePlot R package developed by our and overlaid on the UMAP projection (**Figure 1D**), facilitated the annotation of 21 cell types through the expression of canonical gene markers. Pan-fibroblasts were identified by *Pdgfra* and *Dpt (* 19), with reticular fibroblasts marked by *Pcolce2* and *Aebp1 (* 20), papillary fibroblasts by *Tmeff2* and *Gpc3 (* 21), and perivascular fibroblasts by *Tnmd* and *Lama2 (* 20). Keratinocyte subtypes were characterized by pan-keratinocytes marked by *Krt5* and *Krt14*, spinous keratinocytes by *Krt1* and *Mt4 (* 22), suprabasal keratinocytes by *Alox12e*, *Orm1*, and *Krt10 (* 12), hair follicle keratinocytes (HFH-I: *Lgr5*; HFK-II: *Vsnl1*, *Krt79*) *(* 12), proliferating keratinocytes by *Mki67 (* 23), granular layer keratinocytes by *Akr1c18*, and basal keratinocytes by *Pard6b (* 12). Immune cells were annotated as general cytotoxic T lymphocytes (CTL), comprising various subtypes identified by *Cd3d*, *Cd3e*, and *Cd3g* (pan-T cell markers) (24) along with *Nkg7* (cytotoxic activity marker) (25), including activating T cells marked by *Il2rb* (26), effector T cells marked by *Gzmb* (27), and exhausted T cells marked by *Lag3 (* 28). Additional cell types included endothelial cells (*Pecam1*, *Cdh5*) (29), smooth muscle cells (*Myoz1*, *Tcap*) (30), melanocytes (*Rapsn*) (31, 32), and vascular smooth muscle cells (*Lmod1*, *Pcp4l1*) *(* 33, 34), confirming the diversity of cellular populations in the dataset.

Differential gene expression analysis revealed distinct transcriptional profiles between wounded and control conditions across cell clusters, as shown in a heatmap of differentially expressed genes (DEGs) (**Figure 1E**). Notably, the upregulation of *Epcam* in suprabasal keratinocytes (SBK) and *Cdh5* and *Pecam1* in endothelial cells (EC) suggests enhanced epithelial integrity and angiogenesis respectively (35, 36), consistent with the transition to the proliferation phase. Changes in cellular composition were quantified in a stacked bar plot (**Figure 1F**), with detailed abundance data provided in **Supplementary Table 1**. For instance, papillary fibroblasts (PF) increased from 0.71% in control to 11.82% in wounded skin, while classical monocytes rose from 0.48% to 11.27%. Conversely, hair follicle keratinocytes (HFK-I) decreased from 16.81% to 1.43% in wounded skin, reflecting dynamic shifts in cellular populations during wound healing.

Cell-cell interactions were analyzed to elucidate intercellular communication networks. Bar plots (**Figure 1G**) summarized the overall number and strength of interactions, revealing a significant increase in both metrics in wounded skin compared to control. Circular plots (**Figure 1H**) highlighted differential interaction strengths between specific cell types, such as enhanced communication between M1 macrophages and neutrophils in wounded skin, potentially driven by inflammatory signaling pathways [insert specific ligand-receptor pairs, if analyzed]. These findings underscore the complex cellular and molecular dynamics orchestrating epidermal repair, particularly the prominent role of immune cells like neutrophils, classical monocytes, and M1 macrophages in the wound environment.

### Chemotactic signal analysis of phagocytes in wounded skin

**Figure 2:**
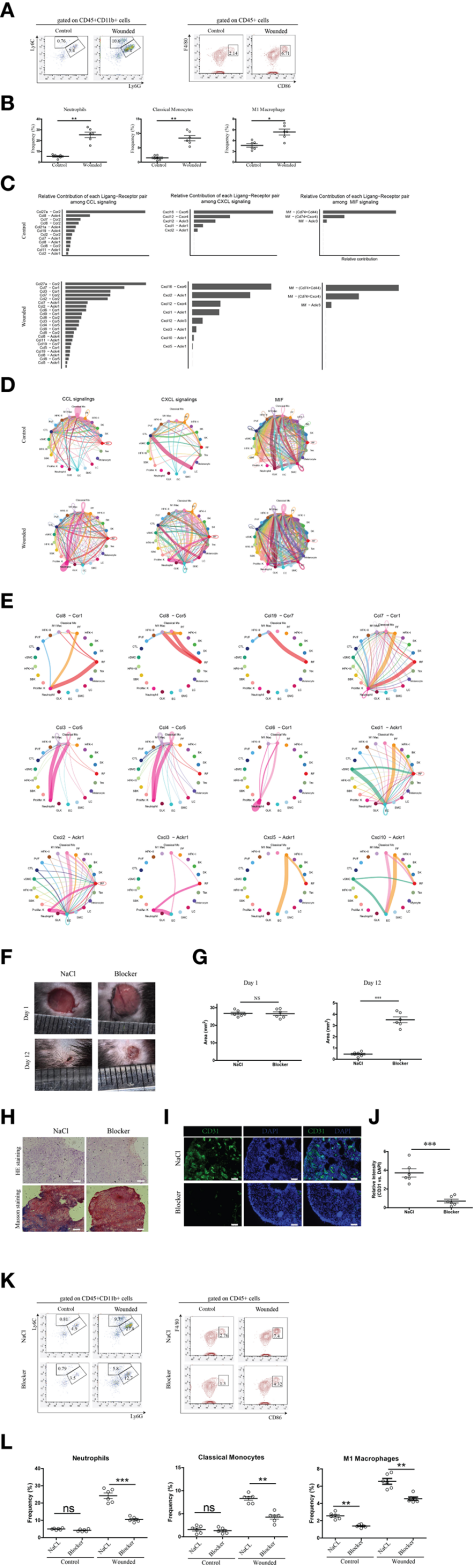
Chemotactic signal analysis of neutrophils, classical monocytes, and m1 macrophages in wounded skin. **(A)** Representative flow cytometry plots of phagocytes (mainly neutrophils, classical monocytes and M1 macropahges) from control and wounded skin samples, gated on CD45+CD11b+ populations. **(B)** Statistical dot plots comparing the relative frequencies of neutrophils, classical monocytes, and M1 macrophages in control versus wounded skin samples. Each dot corresponds to a single readout from one mouse, six mice in each group. Data are representative of findings from three independent experiments. Statistical significance is denoted as follows: ***p < 0.001. **(C)** Bar graphs depicting the relative contribution of each ligand-receptor pair (e.g., CCL, CXCL) to chemotactic signaling in neutrophils, classical monocytes, and M1 macrophages, comparing control and wounded conditions. **(D)** Circular plots illustrating cell-cell interaction networks and their strengths for Neutrophils, Classical Monocytes, and M1 Macrophages, with comparisons between control and wounded conditions. **(E)** Detailed circular plots showing differential cell-cell interaction strengths and numbers for specific ligand-receptor pairs (e.g., CCL-, CXCL-, CX3CR1-Akt) in wounded biopsies across the different cell types. **(F)** Representative images of skin wounds in mice treated with NaCl (Control) or Blocker at Day 1 and Day 12 post-injury. Scale bars indicate 100 μm. The blocker cocktail comprised BX471 (a CCR1 antagonist), Maraviroc (a CCR5 antagonist), and Blocking Peptide LS-E41165 (an ACKR1 inhibitor). **(G)** Quantitative analysis of wound area (mm²) in mice treated with NaCl or Blocker at Day 1 and Day 12. Each dot corresponds to a single readout from one mouse, six mice in each group. Statistical significance is denoted as follows: NS (not significant), ***p < 0.001. **(H)** Histological assessment of skin samples treated with NaCl or Blocker, stained with Hematoxylin and Eosin **(HE)** and Masson’s trichrome. Scale bars represent 100 µm. **(I)** Immunofluorescence staining of skin samples treated with NaCl or Blocker, showing CD31 (green) and DAPI (blue) staining. Scale bars represent 50 µm. **(J)** Quantitative analysis of relative CD31 versus DAPI intensity in skin samples treated with NaCl or Blocker. Each dot corresponds to a single readout from one mouse, six mice in each group. Data are representative of findings from two independent experiments. Statistical significance is denoted as follows: ***p < 0.001. **(K)** Representative flow cytometry plots illustrating the reduced frequencies of neutrophils, classical monocytes, and M1 macrophages in wounded skin following administration of a cocktail, compared to controls injected with saline. **(L)** Statistical dot plots comparing the frequency percentages of neutrophils, classical monocytes, and M1 macrophages between control and wounded skin samples, with statistical significance indicated (ns = not significant, *p<0.05, **p < 0.01, ***p < 0.001). Each dot corresponds to a single readout from one mouse, six mice in each group. Data are representative of findings from three independent experiments.

Given that biopsies were isolated four days post-wounding, a time point corresponding to the late inflammation phase or the transition to the proliferation phase of wound healing (2, 7), we conducted downstream analysis to explore the chemotactic signals driving phagocyte recruitment. This investigation validated the drastic increase in neutrophils, classical monocytes, and M1 macrophages observed in the scRNA-seq data (**Figure 1**), as these phagocytes play a critical role in removing debris and dead tissues, thereby speeding wound healing and enhancing tissue repair quality (37). Flow cytometry of CD45+CD11b+ phagocyte populations from control and wounded skin samples confirmed elevated frequencies in wounded conditions (**Figure 2A**), a finding substantiated by statistical dot plots showing significant differences in relative frequencies between control and wounded skin, with each dot representing an individual readout from three independent experiments (**Figure 2B**). To elucidate the mechanisms underlying this infiltration, we performed a detailed chemotactic signaling analysis, revealing distinct contributions of ligand-receptor pairs such as CCL and CXCL, with marked enhancement in wounded skin across all three phagocyte types, as depicted in bar graphs (**Figure 2C**). Circular plots illustrated expanded cell-cell interaction networks and increased interaction strengths for these phagocytes in wounded conditions compared to controls (**Figure 2D**), with detailed circular plots (**Figure 2E**) highlighting differential strengths for specific pairs, including those induced by CCR5, CCR1, and ACKR1. Specifically, CCR5 signaling, mediated by *CCL3* and *CCL5*, was prominently associated with classical monocyte and M1 macrophage infiltration (38), while CCR1 signaling, driven by *CCL3* and *CCL7*, facilitated neutrophil and monocyte recruitment. ACKR1 signaling, involving *CXCL1* and *CXCL2*, further supported neutrophil infiltration by scavenging chemokines at the wound site. In intervention experiments, administration of a cocktail comprising BX471 (a CCR1 antagonist) (39), Maraviroc (a CCR5 antagonist) (40), and Blocking Peptide LS-E41165 (an ACKR1 inhibitor) was performed. The experiment investigated the effects of inhibition of CCR1, CCR5, and ACKR1 on phagocyte infiltration and subsequent wound healing outcomes in murine models. Representative images of skin wounds treated with NaCl (control) or Blocker (inhibitor) at Day 1 and Day 12 post-injury (**Figure 2F**) revealed a noticeable bigger area of wound size in the Blocker-treated group by Day 12. Quantitative analysis of wound area (**Figure 2G**) showed no significant difference at Day 1, but significantly different at Day 12, indicating delayed wound closure.

Histological assessment using Hematoxylin and Eosin (HE) and Masson’s trichrome staining (**Figure 2H**) of peri-wound skin samples at Day 12 demonstrated reduced re-epithelialization and extracellular matrix (ECM) remodeling in the Blocker-treated group compared to NaCl. Immunofluorescence staining for CD31 (green) and DAPI (blue) (**Figure 2I**) highlighted decreased vascularization in the Blocker group, with quantitative analysis (**Figure 2J**) showing a significant reduction in relative CD31 versus DAPI intensity. Additionally, Ki-67 immunofloroscent (data not shown) confirmed reduced cell proliferation in the Blocker-treated samples.

Flow cytometry analysis of CD45+CD11b- and CD45+ cell populations (**Figures 2K, L**) further supported reduced phagocyte infiltration. In the CD45+CD11b+ gate, Ly6G+Ly6C- neutrophil frequencies decreased significantly, and in the CD45+ gate, CD86+ cell frequencies dropped obviously. These data collectively indicate that inhibition of CCR/ACKR1 signaling not only reduces immune cell infiltration but also affects angiogenesis, and ECM remodeling, underscoring a mechanistic role in wound healing outcomes.

### Comprehensive analysis of cell-cell interaction dynamics and signaling pathways

**Figure 3:**
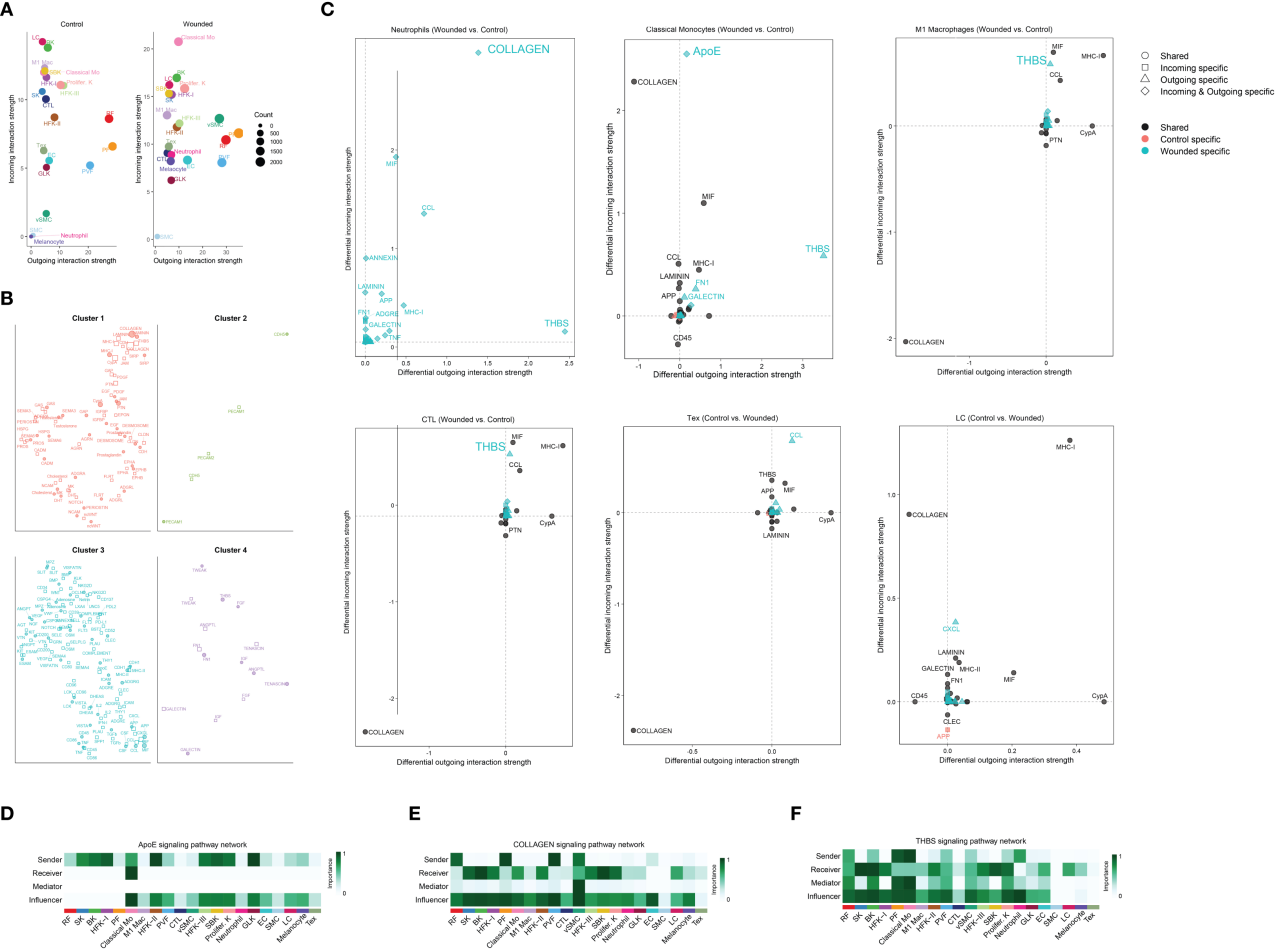
Comprehensive analysis of cell-cell interaction dynamics and signaling pathways in wound healing microenvironment via cellchat. **(A)** Scatter plot comparing the incoming and outgoing interaction strengths of various cell types between control and wounded skin samples, as determined by CellChat analysis. Cell types include Langerhans cells (LC), basal keratinocytes (BK), M1 macrophages (M1 Mac), suprabasal keratinocytes (SBK), proliferating keratinocytes (Prolifer. K), spinous keratinocytes (SK), cytotoxic T lymphocytes (CTL), reticular fibroblasts (RF), hair follicle keratinocytes I and II (HFK-I, HFK-II), hair follicle keratinocytes III (HFK-III), effector T cells (Tex), endothelial cells (EC), granular layer keratinocytes (GLK), perivascular fibroblasts (PVF), melanocytes, vascular smooth muscle cells (VSMC), smooth muscle cells (SMC), and classical monocytes (Classical Mo). Interaction strengths are scaled by count, with circle sizes corresponding to cell abundance (0 to 2000 cells). **(B)** Network plots illustrating the ligand-receptor interactions or signaling molecules within four distinct cell clusters, as determined by CellChat analysis. Cluster 1 features interactions involving collagen, laminin, MHC molecules, prostaglandins, and cadherins; Cluster 2 highlights CDH5 and PECAM1; Cluster 3 includes MPZ, visfatin, slit proteins, and angiopoietins; and Cluster 4 is enriched with TWEAK, tenascin, galectin, and IGF. Node sizes and connections represent the relative abundance and interaction strengths of the molecules, respectively. **(C)** Scatter plots illustrating differential incoming and outgoing interaction strengths of ligand-receptor pairs between wounded and control skin samples, as determined by CellChat analysis. Panels depict: (1) Neutrophils, with key ligands including COLLAGEN and CCL; (2) Classical Monocytes, with APOE and COLLAGEN; (3) M1 Macrophages, with THBS, MHC-II, and CYPA; (4) Cytotoxic T Lymphocytes (CTL), with THBS, CCL, and MHC-II; (5) Effector T cells (Tex), with COLLAGEN, CYPA, and APP; and (6) Langerhans Cells (LC), with COLLAGEN, LAMININ, and CLEC. Symbols indicate shared (○), incoming-specific (□), outgoing-specific (△), incoming and outgoing-specific (⋄), control-specific (•), and wounded-specific (▴) interactions, with statistical significance inferred from differential strengths. **(D)** Heatmap illustrating the ApoE signaling pathway network, depicting the importance of sender, receiver, mediator, and influencer roles across cell. Importance is scaled from 0 (low) to 1 (high). **(E)** Heatmap illustrating the COLLAGEN signaling pathway network, highlighting the importance of sender, receiver, mediator, and influencer roles across the same cell types, with importance scaled from 0 (low) to 1 (high). **(F)** Heatmap illustrating the THBS signaling pathway network, showing the importance of sender, receiver, mediator, and influencer roles across the same cell types, with importance scaled from 0 (low) to 1 (high).

To further characterize the wound healing microenvironment, we analyzed cell-cell interaction dynamics and signaling pathways across the 21 identified cell types during the late inflammatory phase. A scatter plot compared the incoming and outgoing interaction strengths between control and wounded skin samples, revealing dynamic changes in interaction activity for various cell types (**Figure 3A**, **Supplementary Tables 2**, **3**). Network plots delineated the overall increase in signaling strength in wounded skin, classifying interactions into four clusters based on their functional roles: Cluster 1, encompassing collagen, laminin, MHC molecules, prostaglandins, and cadherins, was classified as extracellular matrix (ECM) and immune modulation signaling, reflecting roles in tissue structure and immune regulation; Cluster 2, featuring *CDH5* and *PECAM1*, was categorized as vascular adhesion signaling, indicative of endothelial cell interactions and angiogenesis; Cluster 3, including *MPZ*, visfatin, slit proteins, and angiopoietins, was identified as neurovascular and growth factor signaling, supporting nerve regeneration and vascular development; and Cluster 4, enriched with TWEAK, tenascin, galectin, and IGF, was designated as inflammatory and tissue remodeling signaling, associated with inflammation resolution and matrix reorganization (**Figure 3B**). Focused analysis of immune cell signaling changes showed differential incoming and outgoing interaction strengths for ligand-receptor pairs in neutrophils (*COLLAGEN*, *CCL*), classical monocytes (*APOE*, *COLLAGEN*), M1 macrophages (*THBS*, MHC-II, *CYPA*), cytotoxic T lymphocytes (CTL) (*THBS*, *CCL*, MHC-II), effector T cells (Tex) (*COLLAGEN*, *CYPA*, *APP*), and Langerhans cells (LC) (*COLLAGEN*, *LAMININ*, *CLEC*), with symbols indicating interaction specificity and statistical significance inferred from differential strengths (**Figure 3C**). Heatmaps further detailed the *ApoE*, *COLLAGEN*, and *THBS* signaling pathway networks, highlighting the importance of sender, receiver, mediator, and influencer roles across cell types, with importance scaled from 0 (low) to 1 (high) (**Figures 3D–F**). These analyses provide a comprehensive view of the intercellular communication landscape, linking immune cell infiltration to key signaling pathways that support wound repair during the late inflammation to early proliferation transition.

### Comparative analysis of VEGF and EGF signaling pathways

The decision to analyze vascular endothelial growth factor (VEGF) and epidermal growth factor (EGF) signaling during the late inflammation phase, corresponding to 4 days post-wounding in our study, reflects the dynamic and overlapping nature of wound healing phases in the mouse model. While VEGF and EGF signaling are traditionally considered critical during the proliferation phase—where angiogenesis (driven by VEGF) and re-epithelialization (driven by EGF) are most active—the late inflammation phase, spanning days 3–5, serves as a transitional period where these processes begin to emerge. At this juncture, the resolution of acute inflammation, marked by phagocyte activity (e.g., neutrophils, classical monocytes, and M1 macrophages as validated in **Figures 1**, **2**), overlaps with the initiation of proliferative activities. The increased cellular composition shifts, such as the rise in papillary fibroblasts and keratinocytes (**Figure 1F**), and the enhanced cell-cell interactions (**Figure 1H**), suggest that preparatory molecular signals, including VEGF and EGF, are already being upregulated to support the impending proliferation phase.

**Figure 4:**
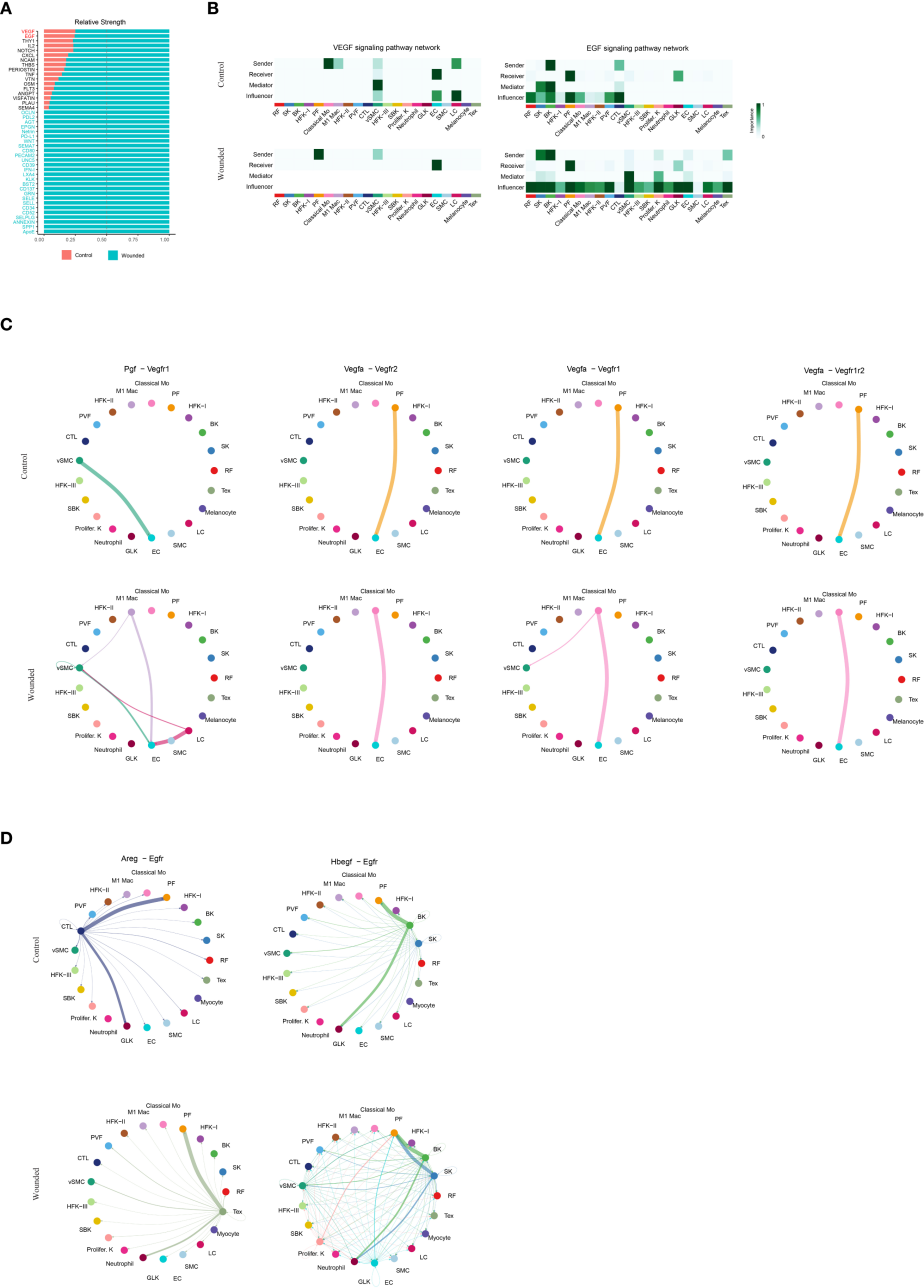
Comparative Analysis of VEGF and EGF Signaling Pathways in Wound Healing Microenvironment. **(A)**. Bar plot illustrating the relative strengths of key ligands. **(B)**. Heatmaps illustrating the VEGF and EGF signaling pathway networks, as determined by CellChat analysis, depicting the importance of sender, receiver, mediator, and influencer roles across cell types. Importance is scaled from 0 (low, light color) to 1 (high, dark color), with separate panels for control and wounded states. **(C)**. Circular plots illustrating cell-cell interaction networks for the Pgf-Vegfr1, Vegfa-Vegfr2, Vegfa-Vegfr1 and Vegfa-Vegfr1r2 signaling pathways, as determined by CellChat analysis, comparing control and wounded skin samples. **(D)**.Circular plots illustrating cell-cell interaction networks for the Areg-Egfr and Hbegf-Egfr signaling pathways, as determined by CellChat analysis, comparing control and wounded skin samples.

In our study, the analysis of VEGF and EGF signaling at this stage is justified by the need to elucidate the molecular priming that facilitates the transition from inflammation to proliferation. The elevated signaling strengths of VEGF and EGF in wounded tissues compared to controls (**Figures 4A**, **B**) indicate that endothelial genesis and re-epithelialization are initiated during late inflammation, driven by pathways such as *Pgf*-*Vegfr1*, *Vegfa*-*Vegfr2*, and *Areg*-*Egfr* (**Figures 4C, D**). This early activation is consistent with the role of immune cells, such as M1 macrophages, in secreting growth factors to stimulate angiogenesis and epithelial repair, as observed in the CellChat analysis (**Figure 3C**). Furthermore, the differential expression of endothelial genesis- and re-epithelialization-related factors (**Supplementary Figure 3**) supports the hypothesis that these signaling pathways are primed during late inflammation to ensure a seamless progression into the proliferation phase. Thus, analyzing VEGF and EGF signaling at this transitional time point provides critical insights into the molecular mechanisms bridging inflammation and proliferation, enhancing our understanding of their coordinated roles in optimizing wound healing outcomes.

## Discussion

The exploration of chemotactic and growth factor signaling during the transition from the inflammation to the proliferation phase of wound healing is of paramount importance, as this period represents a critical juncture that dictates the efficacy and quality of tissue repair (41, 42). The inflammation phase, characterized by the infiltration of immune cells such as neutrophils, classical monocytes, and M1 macrophages, is essential for debris clearance and pathogen defense (43); however, its resolution and seamless progression into the proliferation phase—where angiogenesis and re-epithelialization drive tissue regeneration—require precise molecular coordination. This transitional phase, particularly at 4 days post-injury in the mouse skin model, is marked by a dynamic shift in cellular composition and intercellular communication (44), as evidenced by the alteration of papillary fibroblasts and keratinocytes observed in this study. Chemotactic signaling, mediated by receptors such as CCR5, CCR1, and ACKR1, orchestrates the recruitment and spatial organization of phagocytes, ensuring effective inflammation resolution and setting the stage for proliferative activities. Concurrently, growth factors like vascular endothelial growth factor (VEGF) and epidermal growth factor (EGF), which initiate angiogenesis and re-epithelialization, respectively, are primed during this phase, suggesting a preparatory molecular landscape that bridges the two stages. Understanding these signaling pathways is essential not only to elucidate the mechanisms underlying this transition but also to identify potential intervention manner, as demonstrated by the significant reduction of phagocyte infiltration with antagonist cocktails. Failure to regulate this transition can lead to chronic wounds or excessive scarring, underscoring the necessity of investigating these processes to optimize wound healing outcomes.

The discovery of significant phagocyte accumulation, including neutrophils, classical monocytes, and M1 macrophages, during the transition from inflammation to proliferation at 4 days post-wounding in this study contrasts with and extends the findings of previous investigations delineating the inflammation and proliferation stages. Previous studies have established that the inflammation phase (typically days 0–3 in mouse models) is characterized by a robust influx of phagocytes, with neutrophils peaking early to eliminate pathogens and debris, followed by monocytes differentiating into macrophages to continue clearance and initiate cytokine release (45). This phase is marked by a transient peak, with phagocyte numbers declining as inflammation resolves. In contrast, the proliferation phase (days 5–14) is traditionally associated with reduced phagocyte presence (46), as fibroblasts, keratinocytes, and endothelial cells dominate to support angiogenesis and re-epithelialization, with macrophages shifting toward an M2 phenotype to promote tissue repair. This study, however, reveals a sustained and enhanced accumulation of phagocytes at the transitional 4-day mark, validated by a rise from 0.48% to 11.27% for classical monocytes and corroborated by flow cytometry, suggesting a prolonged inflammatory role that overlaps with proliferative initiation. This finding indicates that the transition phase serves as a critical bridge, where chemotactic signaling via CCR5, CCR1, and ACKR1 sustains phagocyte activity to facilitate debris clearance while priming the microenvironment for subsequent regenerative processes, a dynamic not fully captured in the discrete staging of prior research.

The discovery of elevated signaling through the VEGF pathways (Pgf-Vegfr1, Vegfa-Vegfr2) (47, 48) and EGF pathways (Areg-Egfr, Hbegf-Egfr) (49, 50) during the late inflammation phase at 4 days post-wounding underscores their critical role in bridging inflammation and proliferation in wound healing. Traditionally, VEGF signaling, mediated by ligands such as Pgf and Vegfa binding to their receptors Vegfr1 and Vegfr2 (47), is recognized as a primary driver of angiogenesis during the proliferation phase, promoting endothelial cell proliferation and vascular network formation. Similarly, EGF signaling, through ligands Areg and Hbegf interacting with Egfr (49, 50), is well-established for stimulating keratinocyte proliferation and migration, essential for re-epithelialization. This study reveals their early activation in the transitional phase, with increased signaling strengths in wounded mouse skin compared to controls (**Figure 4A**), suggesting a preparatory molecular priming that anticipates the proliferative demands. This finding is particularly significant, as it indicates that immune cells, such as M1 macrophages, may initiate these pathways by secreting growth factors, facilitating a seamless transition from inflammation resolution to tissue regeneration. The identification of these pathways at this juncture offers valuable insights into the temporal coordination of wound repair and highlights potential intervention manner. Modulating VEGF and EGF signaling could enhance angiogenesis and re-epithelialization in impaired healing conditions, such as chronic wounds, thereby improving clinical outcomes.

A key contribution of this study is the development and introduction of the R package OptiRes, designed to objectively identify the optimal clustering resolution in single-cell RNA sequencing data. Determining suitable resolution parameters is a critical step in accurately delineating cell populations, yet it remains a challenge due to the subjective nature of conventional methods, which often rely on visual inspection or arbitrary choices. OptiRes addresses this gap by systematically calculating the silhouette score across multiple resolutions, providing a quantitative metric to guide clustering decisions. This approach enhances the robustness, reproducibility, and precision of cell type identification, which is fundamental for subsequent analyses such as cell composition profiling and intercellular communication mapping. By integrating OptiRes into the analytical pipeline, researchers can confidently select the most biologically meaningful cluster resolutions, ultimately leading to more accurate interpretations of cellular heterogeneity and signaling dynamics during complex biological processes like wound healing.

This study, while providing novel insights into the transitional phase of wound healing, is subject to several limitations. First, the analysis is based on a single time point (4 days post-wounding), which may not fully capture the dynamic progression of cellular and molecular events across the late inflammation to proliferation transition. The reliance on scRNA-seq data from NCBI GEO (GSE142471) introduces potential variability due to differences in sample preparation or sequencing depth, which could affect the robustness of cell type identification and gene expression profiles. Additionally, the intervention with the antagonist cocktail (BX471, Maraviroc, Blocking Peptide LS-E41165) was conducted over a limited duration (3 days), potentially underestimating long-term effects on phagocyte dynamics or tissue repair outcomes. The study’s focus on a mouse model may also limit translational relevance to human wound healing, given species-specific differences in immune responses and healing kinetics. Finally, the lack of functional validation for the observed VEGF and EGF pathway activations restricts the ability to confirm their causal roles in priming angiogenesis and re-epithelialization.

## Conclusion

This study provides a comprehensive characterization of the cellular and molecular dynamics during the transition from inflammation to proliferation in wound healing, utilizing single-cell RNA sequencing of mouse skin biopsies at 4 days post-injury. The identification of 21 distinct cell types, facilitated by the OptiRes and TidyGenePlot R packages, revealed significant phagocyte accumulation, including neutrophils, classical monocytes, and M1 macrophages, driven by enhanced chemotactic signaling via CCR5, CCR1, and ACKR1. Additionally, the early activation of VEGF (Pgf-Vegfr1, Vegfa-Vegfr2) and EGF (Areg-Egfr, Hbegf-Egfr) pathways underscores their role in priming angiogenesis and re-epithelialization. The successful reduction of phagocyte infiltration with a cocktail (BX471, Maraviroc, Blocking Peptide LS-E41165) highlights the potential for targeted interventions. These findings elucidate the intercellular communication landscape and molecular mechanisms orchestrating this critical phase, offering insights into optimizing wound repair. Future research should focus on longitudinal analyses and functional validation to translate these discoveries into clinical applications for improved healing outcomes.

## Data Availability

The scRNA-seq dataset analyzed in this study is publicly available in the NCBI Gene Expression Omnibus (GEO) under accession number GSE142471.
