# Supplementary figures and images for: Bridging inflammation and proliferation: scRNA-seq analysis of chemotactic and growth factor signaling in mouse skin wound repair
Source: Front Immunol. 2025 Sep 25;16:1654043. doi: 10.3389/fimmu.2025.1654043 (PMC12507807; doi:10.3389/fimmu.2025.1654043)

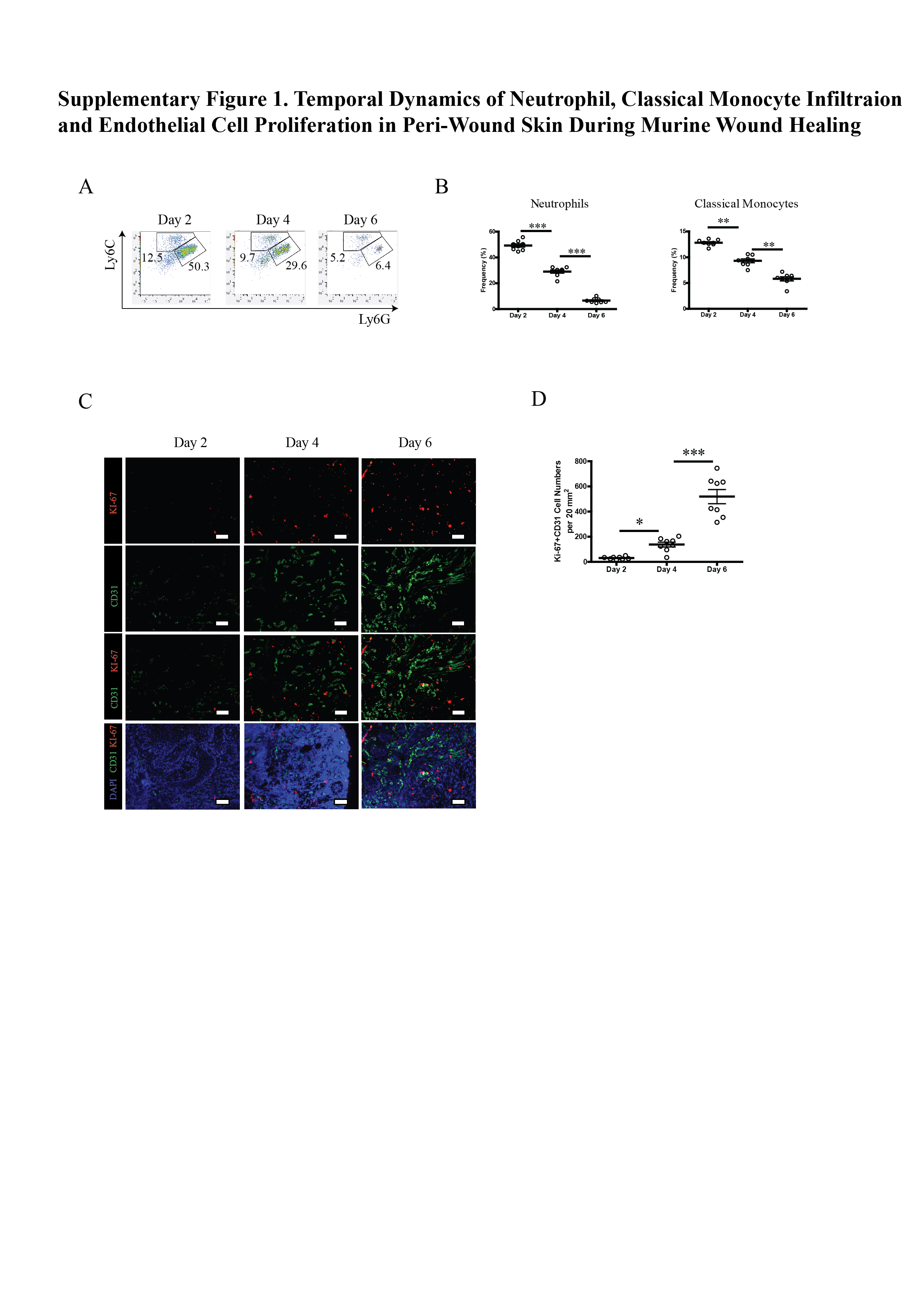

Supplement: Supplementary file 1 [file Image1.jpeg]

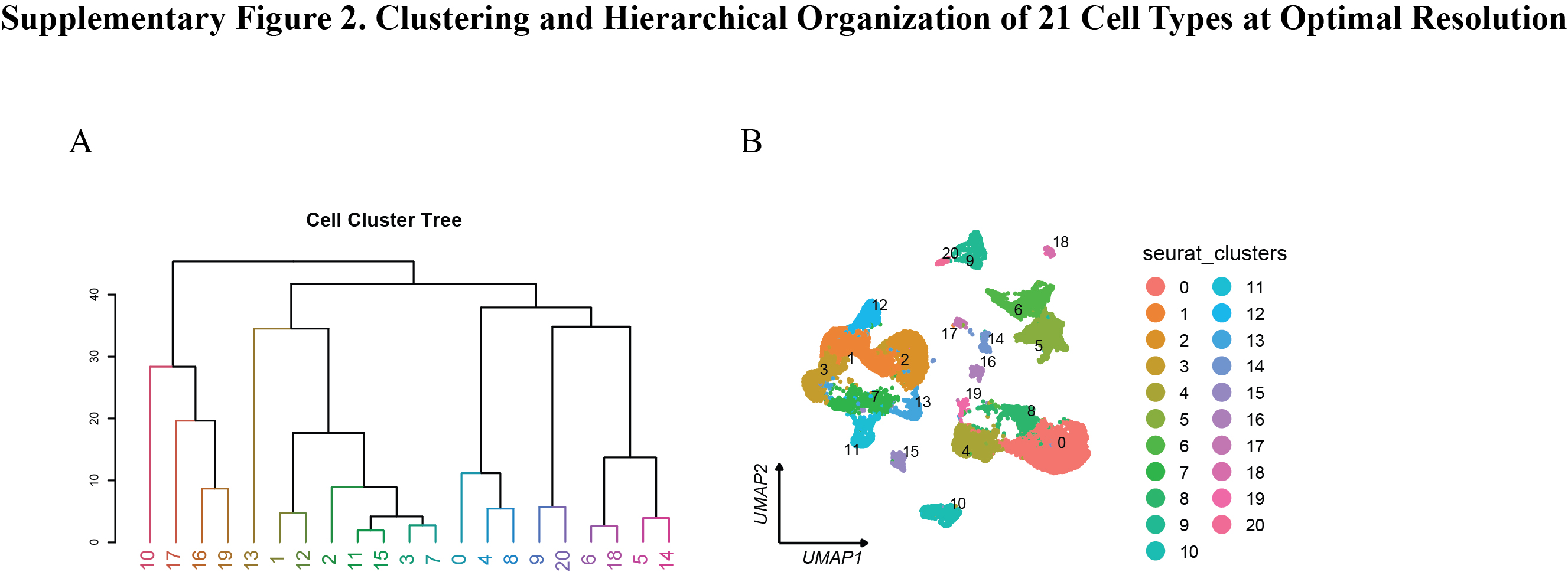

Supplement: Supplementary file 2 [file Image2.jpeg]

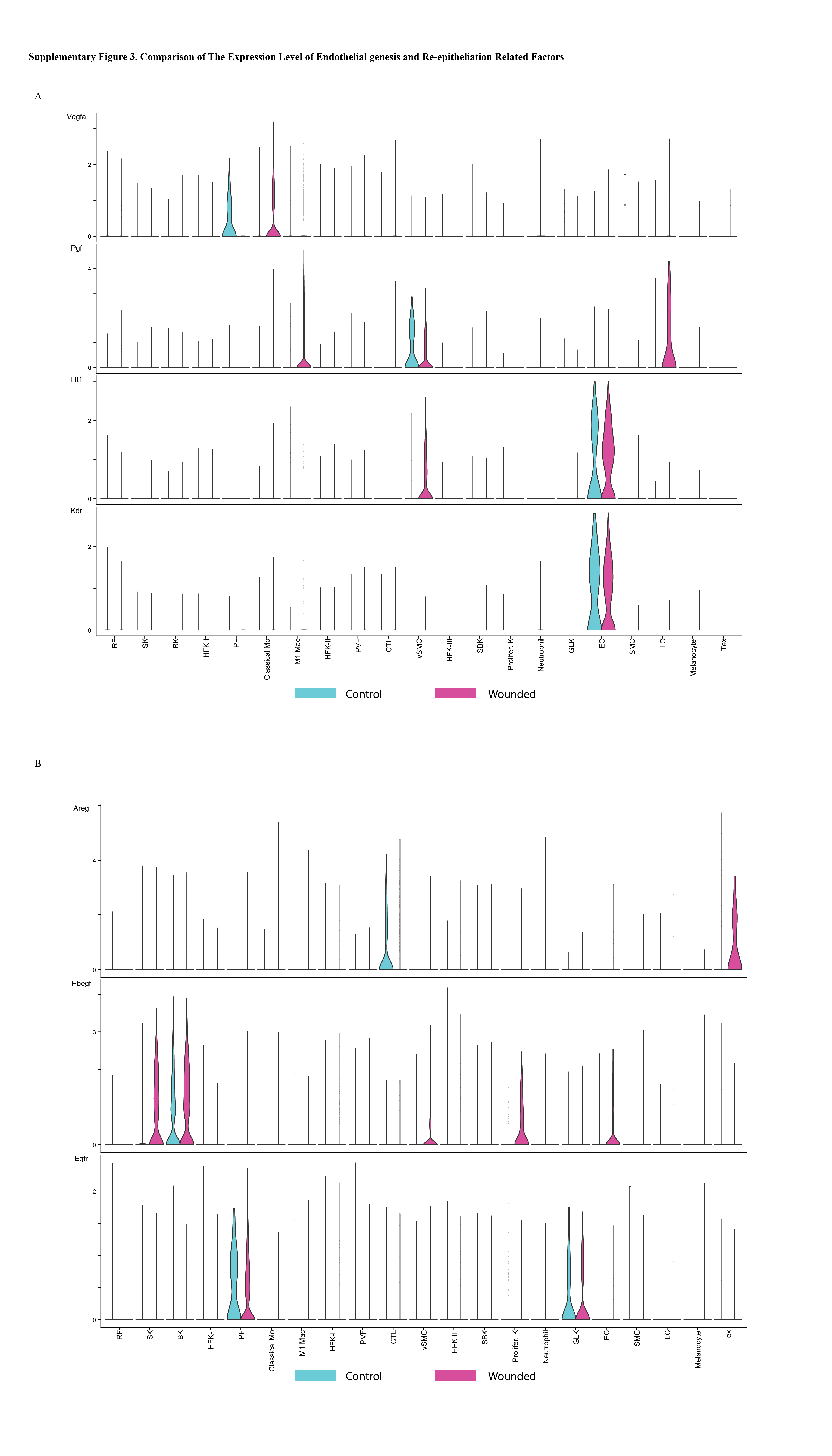

Supplement: Supplementary file 3 [file Image3.jpeg]
